# Cross-domain few-shot learning based on pseudo-Siamese neural network

**DOI:** 10.1038/s41598-023-28588-y

**Published:** 2023-01-25

**Authors:** Yuxuan Gong, Yuqi Yue, Weidong Ji, Guohui Zhou

**Affiliations:** School of Computer Science and Information Engineering, Harbin Normal University, Harbin, 150025 Heilongjiang People’s Republic of China

**Keywords:** Mathematics and computing, Computer science

## Abstract

Cross-domain few-shot learning is one of the research highlights in machine learning. The difficulty lies in the accuracy drop of cross-domain network learning on a single domain due to the differences between the domains. To alleviate the problem, according to the idea of contour cognition and the process of human recognition, we propose a few-shot learning method based on pseudo-Siamese convolution neural network. The original image and the sketch map are respectively sent to the branch network in the pre-training and meta-learning process. While maintaining the original image features, the contour features are separately extracted as branch for training at the same time to improve the accuracy and generalization of learning. We conduct cross-domain few-shot learning experiments and good results have been achieved using mini-ImageNet as source domain, EuroSAT and ChestX as the target domains. Also, the results are qualitatively analyzed using a heatmap to verify the feasibility of our method.

## Introduction

Deep learning methods have achieved remarkable results in many fields, and the accuracy is constantly improving. It is often necessary to rely on a large number of labeled training samples for learning^1^ to learn the distribution of category information. The generalization ability of deep learning largely depends on the size and diversity of the training dataset. However, there may be some practical situations when using deep learning to solve problems. For example, it is difficult to collect a large number of samples, such as clinical samples of certain diseases, or to reduce the cost of obtaining samples. It is still worthwhile to address the problem of how to appropriately identify these categories with small sample size.

With a small number of samples, humans tend to generalize samples based on existing knowledge to identify new categories. But in cases where categories differ significantly from one another or prior knowledge, such as diagnostics in dermatology, radiology, or other fields^2^, it is challenging for both human and machine to distinguish between new classes. Scholars have proposed few-shot learning to solve the problem with few samples. Learning to classify with a small number of training samples is the research goal of few-shot learning. A large number of research results have been proposed, such as MAML^3^, RelationNet^4^, MetaOpt^5^, ProtoNet^6^, and so on. Few-shot learning usually has two phases: the meta-learning phase and the meta-testing phase. The meta-learning stage uses a large number of categories to train the network, and there are only a small number of samples under each category. The meta-testing stage uses new classes that have not been trained to tune and evaluate the network learning in the meta-learning stage. However, Chen et al. pointed out in their research that when the basic class domain is quite different from the new class domain, the accuracy of the few-shot learning algorithms based on meta-learning are not as good as that of the traditional pre-training fine-tuning algorithm^7^. Guo et al. proposed a standard for cross-domain few-shot learning and conducted further research on this problem, and found that different meta-learning models perform similarly in the same target domain, and the performance of the same meta-learning model in different target domains is significantly different. As the domain similarity increases, the classification accuracy of the models improves. Some meta-learning methods are even less effective than random weight networks in some cross-domain issues^8^.

Based on the problems above, Zhao et al. defined the domain-adaptive few-shot learning problem (DA-FSL) and proposed a domain-adversarial prototyping network (DAPN) model^9^. By explicitly enhancing the inter-class separation of source/target domains before domain-adaptive feature embedding learning, it can mitigate the negative impact of domain alignment on few-shot learning. Yuan et al. proposed the Bilevel Episode Strategy (BL-ES)^10^. The outer episodes in BL-ES continuously simulate cross-domain few-shot tasks, and the inner episodes learn to drive the inductive graph neural network (IGN) to introduce the common features of the test classes. The correlation between samples is captured by using IGN. Geometric constraints are introduced to the training loss to improve robustness. Li et al. adopted a conditional adversarial domain adaptation strategy to learn a domain-adaptive feature embedding space^11^. It aims to achieve domain distribution alignment to address the cross-domain problem in hyperspectral image classification. Lu et al. proposed the Domain Alignment Prototype Network (DA-PN) to handle the cross-domain few-shot recognition task, and designed a domain alignment module to minimize the maximum average difference between the training dataset and the test dataset in the feature space^12^. Liu et al. proposed a geometric algebra graph neural network (GA-GNN) as the metric network for cross-domain few-shot classification tasks^13^. The feature nodes are mapped into the hyper-complex vector to reduce the distortion of feature information with the increased hidden layers. Tian et al. proposed a momentum memory contrastive few-shot learning method based on the distance metric and transfer learning^14^. The method adopts an external memory bank and a contrastive loss function to constrain the feature representation of the samples in training.

The methods above achieve cross-domain learning by aligning the source domain with the target domain. Most of them project the target domain and the source domain into the same metric space, and calculate the relative distance of the samples for classification. However, there are few studies on using the domain-independent information in the samples themselves in cross-domain few-shot learning. The evaluation benchmarks CUB^15^, Omniglot^16^, CIFAR-FS^17^ and tieredImageNet^18^ are more extensive and comprehensive than previous benchmarks, but still limited to natural and undistorted images. Inspired by the fact that people can easily identify images of sketches or images with only contour features, we imitate the process of human recognition and classification based on the idea of cognitive contours. That is, people often recognize objects according to their general outline features, and then judge the classification of objects according to their detailed features. We choose the sketch maps of the generated samples as domain-independent features. The generated sketch map is different from the grayscale image, which not only reduces the color influence, but also greatly reduces the information except the contour, so as to deal with the difference between domains. A few-shot learning method based on pseudo-Siamese convolutional neural network (Cross-Domain Pseudo-Siamese Network, CDPSN) is proposed. The method first extracts the contour features of the image, imitates the light and shadow display method of the sketch map, and uses the grayscale change to simulate the distance of human vision to reduce the difference between domains. Then the pseudo-Siamese convolutional neural network is used to learn the extracted contour features of the images as a branch while learning the original samples as another branch. Fully connected layers are used to replace the similarity metric calculation process of the pseudo-Siamese convolutional neural network to adaptively adjust the attention range of the preprocessing branch according to the number of samples. We train the model on mini-ImageNet^19^ and test on ChestX^20^ and EuroSAT^21^. These datasets are not limited to natural images and have greater inter-domain differences than those mentioned above.

The main contributions of this paper are summarized as follows:

1. We propose a training method that extracts the contour features of the samples and sends the original samples and the extracted features to the two branches of the neural network separately. Use the sketch map generation method as a preprocessing process to reduce the difference between the domains.
2. We introduce the pseudo-Siamese convolutional neural network structure to deal with few-shot cross-domain problems with large differences between source and target domains. The dual-input structure of the pseudo-Siamese neural network can learn different samples separately. With keeping the original feature extraction process unchanged, a branch network with the extracted image contour as input is added to improve the generalization ability.
3. For the network proposed in this paper, the heatmap analysis of the pre-training and meta-learning process is carried out to intuitively illustrate the feasibility of the network.

## Related works

### Few-shot learning

Few-shot learning aims to identify new classes with a small number of samples. Few-shot learning mostly uses the meta-learning approach, which can be broadly divided into three categories: (1) Model-based few-shot learning. A model trained on the source classes is fine-tuned and then quickly adapted to the target classes^22,23^. (2) Metric-based few-shot learning. This category often uses the nearest neighbor search method. The prototype network (ProtoNet)^6^ learns a metric space to classify samples by computing the distance between the test samples and the prototype representation of each target class, and is able to utilize unlabeled samples through improvements. MatchingNet^19^ builds different encoders for the support set and query set respectively. RelationNet^4^ classifies samples of the target class by computing the relationship score between the query samples and samples of each new class. (3) Optimization-based few-shot learning. Different from the traditional optimization algorithm using gradient descent, this kind of few-shot learning uses a new optimization algorithm to adapt to the small sample size, such as the MAML algorithm^3^ and the Meta-Learner LSTM^24^ algorithm. MTL method^25^ designs a new set of convolution kernel scaling parameters. According to the idea of transfer learning, the convolution kernel weights learned in pre-training remain unchanged during the meta-learning process, and the convolution kernel scaling parameters will be re-established and updated in the meta-learning phase. In this paper, based on the idea of transfer learning, the branch structure of the pseudo-Siamese neural network is designed as a complete residual neural network.

### Domain adaptation

Unsupervised Domain Adaptation (UDA) has become an effective mean to solve domain adaptation problems recently. Traditional unsupervised domain adaptation models^26,27^ usually adopt subspace alignment techniques. Modern UDA methods^28–30^ use more adversarial learning to minimize the distance between source and target features through the discriminator. However, even enforcing global domain distribution alignment, it often results in per-class alignment, in which causes the reduce discriminativeness of learned feature representations for few-shot learning tasks. DPDAPN^31^ adopts global domain data distribution alignment. Due to the limited scope of application of domain alignment, DPDAPN is still limited to natural and undistorted images. With this problem, our method directly preprocesses samples to reduce the number of sample features to narrow the difference between domains and enhances the network's ability to adapt to different domains. Additionally, the original image training process is preserved to learn features like color and increase the recognition accuracy.

### Cross-domain few-shot learning

Cross-domain few-shot learning focuses on domain changes. The cross-domain few-shot learning benchmark BSCD-FSL (Broader Study of Cross-Domain Few-Shot Learning)^8^ highlights the impact of inter-sample similarity measures on accuracy. Under the setting of FSDA^32^, the source and target domains share the same set of classes, and there is no large domain variation. In this paper, based on the BSCD-FSL benchmark, the source domain uses the mini-ImageNet dataset, and the datasets EuroSAT and ChestX, which are quite different from the source domain, are used as the source domain. We propose a cross-domain few-shot learning method CDPSN based on pseudo-Siamese neural network. Our method only needs a small number of samples with labels. while DA-based (domain adaptation based) few-shot learning assumes the condition that a large number of unlabeled samples can be obtained from the target domain, which may require larger costs for some domains.

## Method

### Problem definition

The source domain $S$ is used as the training set and the target domain $T$ as the test set. The cross-domain few-shot learning problem can be defined by the following equation

1 $$S = \{ (x_{i} ,y_{i} )\}_{i = 1}^{Ns} ;T = \left\{ {\{ (\hat{x}_{j} ,\hat{y}_{j} )\}_{j = 1}^{Nt} ,\{ \hat{x}_{Nt + 1} ,...,\hat{x}_{Nt + q} \} } \right\}$$

where $x_{i}$ is the sample in the source domain,$y_{i} \in Y_{s}$, $Y_{s} = \{ 1,...,L_{s} \}$ is the corresponding correct label of $x_{i}$. $\hat{x}_{j}$ is a sample from target domain $T$. $\hat{y}_{j} \in Y_{T}$, $Y_{T} = \{ L_{S} + 1,...,L_{S} + L_{T} \}$ is the corresponding correct label of $\hat{x}_{j}$. If a few-shot classification task $\tau$ contains only $K$ labeled samples in each of $N$ unique classes, the task $\tau$ is called a $N$-way $K$-shot classification task. According to the above definition, the source and target domains are disjoint $Y_{s} \cap Y_{T} = \emptyset$, the length of the target set $\left\| {Y_{T} } \right\| = L_{T} = N$, the total test samples $Nt = N \times K$. Assume the source domain $S$ is available during the training phase. Cross-domain few-shot learning aims to learn a classification model based on the source domain $S$, and adjust the model parameters with partial data $\{ (\hat{x}_{j} ,\hat{y}_{j} )\}_{j = 1}^{Nt}$ from the target domain $T$ to fit the problem on the target domain.

### Sample preprocessing

In order to obtain the contour features of the samples $x_{i}$, two sketch map generation methods based on gradient and matrix division are used to process the samples. The result of combining the advantages of the two methods is obtained by taking the minimum value of the two generated maps.

#### Gradient-based sketch map generation method

First, convert the sample into a grayscale image, where the grayscale value of the image is the light and dark change of the image, and the gradient represents the change rate of the grayscale. Extract the $x$-direction gradient $\mathop {grad}\limits_{x} (x_{i} )$ and $y$-direction gradient $\mathop {grad}\limits_{y} (x_{i} )$ of the sample. Use the grayscale change to simulate the depth perception of human vision, and adjust the scale of the gradient with $grad(x_{i} ) = \frac{d}{100}grad(x_{i} )$, $d \in (0,100)$ as needed.

Suppose there is a light source located obliquely above the image, the top-view angle of the light source relative to the image is $\alpha$, and the azimuth angle is $\lambda$, the influence degree of the light source on the three directions of space is shown in Eq. (2) as follows

2 $$\left\{ \begin{gathered} dx = \cos (\alpha ) \times \cos (\lambda ) \hfill \\ dy = \cos (\alpha ) \times \sin (\lambda ) \hfill \\ dz = \sin (\alpha ) \hfill \\ \end{gathered} \right.$$

**Figure 1:**
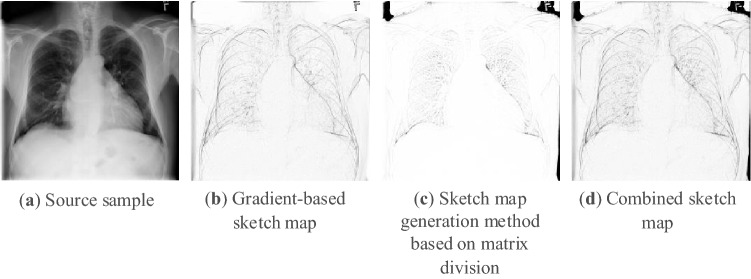
Maps generated by each preprocessing process.

The change of $\alpha$ and $\lambda$ will affect the light and shadow effect of the generated map. The closer $\alpha$ is to $\frac{\pi }{2}$, the less obvious the effect of simulating the distance with the gray value is. The change of $\lambda$ will affect the direction of shadow casting after simulating the distance. The gradient is normalized by Eq. (3) as follows

3 $$\left\{ \begin{gathered} \overline{x} = \frac{{\mathop {grad}\limits_{x} (x_{i} )}}{{\sqrt {\mathop {grad}\limits_{x} (x_{i} )^{2} + \mathop {grad}\limits_{y} (x_{i} )^{2} + 1} }} \hfill \\ \overline{y} = \frac{{\mathop {grad}\limits_{y} (x_{i} )}}{{\sqrt {\mathop {grad}\limits_{x} (x_{i} )^{2} + \mathop {grad}\limits_{y} (x_{i} )^{2} + 1} }} \hfill \\ \overline{z} = \frac{1}{{\sqrt {\mathop {grad}\limits_{x} (x_{i} )^{2} + \mathop {grad}\limits_{y} (x_{i} )^{2} + 1} }} \hfill \\ \end{gathered} \right.$$

where $\overline{x}$, $\overline{y}$, $\overline{z}$ are the normalized gradients. Interacting the light source and the gradient through Eq. (4) and converting the gradient into grayscale to obtain the gradient-based sample contour feature $x_{i}^{grad}$ as follows

4 $$x_{i}^{grad} = 255 \times (dx \times \overline{x} + dy \times \overline{y} + dz \times \overline{z})$$

where $x_{i}^{grad} \in (0,255)$. $dz \times \overline{z}$ acts as a regularization term. Gradient-based sketch map is sensitive to image gradient changes. The assumed light source is a fixed factor used to attenuate the effect of gradients in a single direction, which can reduce the noise. This method can better extract the contour shape. The result of this step is shown in Fig. 1b under the condition of $d = 10$, $\alpha = \frac{\pi }{2.2}$, $\lambda = \frac{\pi }{4}$. For easier viewing, all sketched maps have been reversed. It can be seen that the outline of the gradient generation method is relatively clear, but the details, such as the blood vessels in the picture, are not good enough. The codes of this method can be found in^33^.

#### Sketch map generation method based on matrix division

First, the sample is converted into grayscale images $x_{i}^{gray}$, then apply Gaussian filtering to $x_{i}^{gray}$ Finally, element-wise division is performed between the grayscale image and the Gaussian filtered result. The overall processing process is shown in Eq. (5) as follows

5 $$x_{i}^{divide} = 255 \times divide(x_{i}^{gray} ,Gauss(x_{i}^{gray} ))$$

where $x_{i}^{divide} \in (0,255)$, $divide$ represents element-wise division, and $Gauss$ represents Gaussian filtering. The image after Gaussian blur weakens the gradient of the details, and has less influence on areas with obvious gradient changes. Divide the grayscale images by Gaussian blurred map, which weakens the parts with obvious gradient changes in the grayscale image . This method better highlights the outline of the details in the image. The result of this step is shown in Fig. 1c. It can be seen that the obvious bone outline in the gradient-based sketch map is not clear in this method, and the blood vessel part is clearer than the gradient-based sketch map. We use the OpenCV library for this step.

Combine the results of the two processing methods above to combine the advantages of the two methods by Eq. (6) as follows

6 $$x_{i}^{sk} = \min (x_{i}^{grad} ,x_{i}^{divide} )$$

The combined result is shown in Fig. 1d. The final map not only clearly shows the outline of the bones, but also shows the details of the blood vessels.

### Few-shot learning

First, the feature extractor is trained on large-scale data (such as mini-ImageNet). Second, in the meta-learning stage, the scaling and shifting parameters of the feature extractor are trained so that the feature extractor can quickly adapt to few-shot tasks. Finally, pick the target domain and perform cross-domain meta test.

### Pre-training

Training a feature extractor on large-scale data is similar to the classical pre-training stage. First, a feature extractor $\Theta$ and a classifier $\theta$ are randomly initialized. The pre-processed samples and the original samples are respectively sent to the network branch and optimized by gradient descent. As shown in Eq. (7).

7 $$[\Theta ;\theta ] = :[\Theta ;\theta ] - \gamma \nabla {\mathcal{L}}_{D} ([\Theta ;\theta ])$$

where $D$ is the training set, $\gamma$ is the learning rate, and ${\mathcal{L}}$ represents the empirical loss shown in Eq. (8), e.g., cross-entropy loss.

8 $${\mathcal{L}}_{D} ([\Theta ;\theta ]) = \frac{1}{\left| D \right|}\sum\limits_{(x,y) \in d} {l(f_{[\Theta ;\theta ]} (x),y)}$$

The feature extractor $\Theta$ trained in this stage will participate in the next stage of training. The resulting classifier $\theta$ will be discarded because it is not suitable for subsequent classification tasks.

In this stage, the learning process of the original samples and the samples after processing are carried out separately, and the loss function uses the weighted sum of the two as the loss function of gradient descent, as shown in Eq. (9).

9 $${\mathcal{L}\ominus } = \chi {\mathcal{L}\ominus }_{D} + \delta {\mathcal{L}\ominus }_{Sk}$$

where ${\mathcal{L}\ominus }_{Sk}$ represents the predicted empirical loss of the pre-processed samples. $\chi$ and $\delta$ represent the weight of ${\mathcal{L}\ominus }_{D}$ and ${\mathcal{L}\ominus }_{Sk}$. They control the importance of the two branches in the pre-training process.

**Figure 2:**
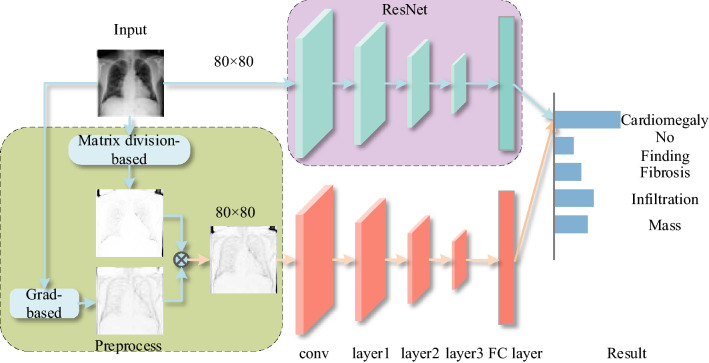
Network structure diagram of the pre-training process.

The pre-training process is shown in Fig. 2. The pseudo-Siamese neural network consists of two networks with the same structure and different weights or two networks with different structures and can input two samples at the same time. The original function is to calculate whether the two images are the same object or whether the image is consistent with the text description. Since the purpose of this method is to classify images rather than compare the similarity of input images, and the pseudo-Siamese network doesn’t output the predicted probability, distance measurement is unnecessary for this task. We alter the distance measurement of the pseudo-Siamese neural network to fully connected layers to combine the results from the two branches and generate the final prediction during the meta-learning phase.

In the pre-training process, the original sample and the preprocessed sample are respectively sent to two residual networks with the same structure but different weights in the pseudo-Siamese neural network for learning, and the prediction results are given by their respective fully connected layers. Calculate the loss according to the loss function, and learn by gradient descent. The two branches in the pseudo-Siamese network relatively independent in the pre-training phase.

### Meta Learning

Due to the large difference between the target domain and the source domain, in the meta-learning stage, according to the given task ${\mathcal{T}}$, the current classifier $\theta^{\prime}$ and feature extractor $\Theta$ are optimized according to the loss of ${\mathcal{T}}^{(tr)}$ by gradient descent.

10 $$[\Theta ;\theta^{\prime}] = :[\Theta ;\theta^{\prime}] - \beta \nabla {\mathcal{L}}_{D} ([\Theta ;\theta^{\prime}])$$

where $\beta$ is the learning rate of meta learning phase. Different from Eq. (7), the new classifier $\theta^{\prime}$ that only concerns a few classes according to the need of task ${\mathcal{T}}$.

Since the feature distance measure of the pseudo-Siamese neural network is not suitable for the cross-domain few-shot learning situation, we use fully connected layers to replace the similarity measure calculation process. The feature matrix obtained from the preprocessed samples after feature extraction will be merged into the feature matrix of the original sample feature extraction as an augmented matrix, and classified through the fully connected layers. The prediction result $\hat{y}$ is expressed as follows

11 $$\hat{y} = F\left( {\left[ {f_{{[\Theta ;\theta^{\prime}]}} (x),f_{{[\Theta_{sk} ;\theta^{\prime}_{sk} ]}} (x_{sk} )} \right]} \right)$$

where $F$ denotes the prediction process of the fully connected layers that fuses two branches. $x_{sk}$ denotes the preprocessed sample. $\theta^{\prime}_{sk}$ denotes the classifier of sketched branch. The loss in this phase is shown as follows

12 $${\mathcal{L}}\left( {[\Theta ;\theta^{\prime}],[\Theta_{sk} ;\theta^{\prime}_{sk} ]} \right) = \frac{1}{\left| D \right|}\sum\limits_{(x,y) \in d} {l(\hat{y},y)}$$

**Figure 3:**
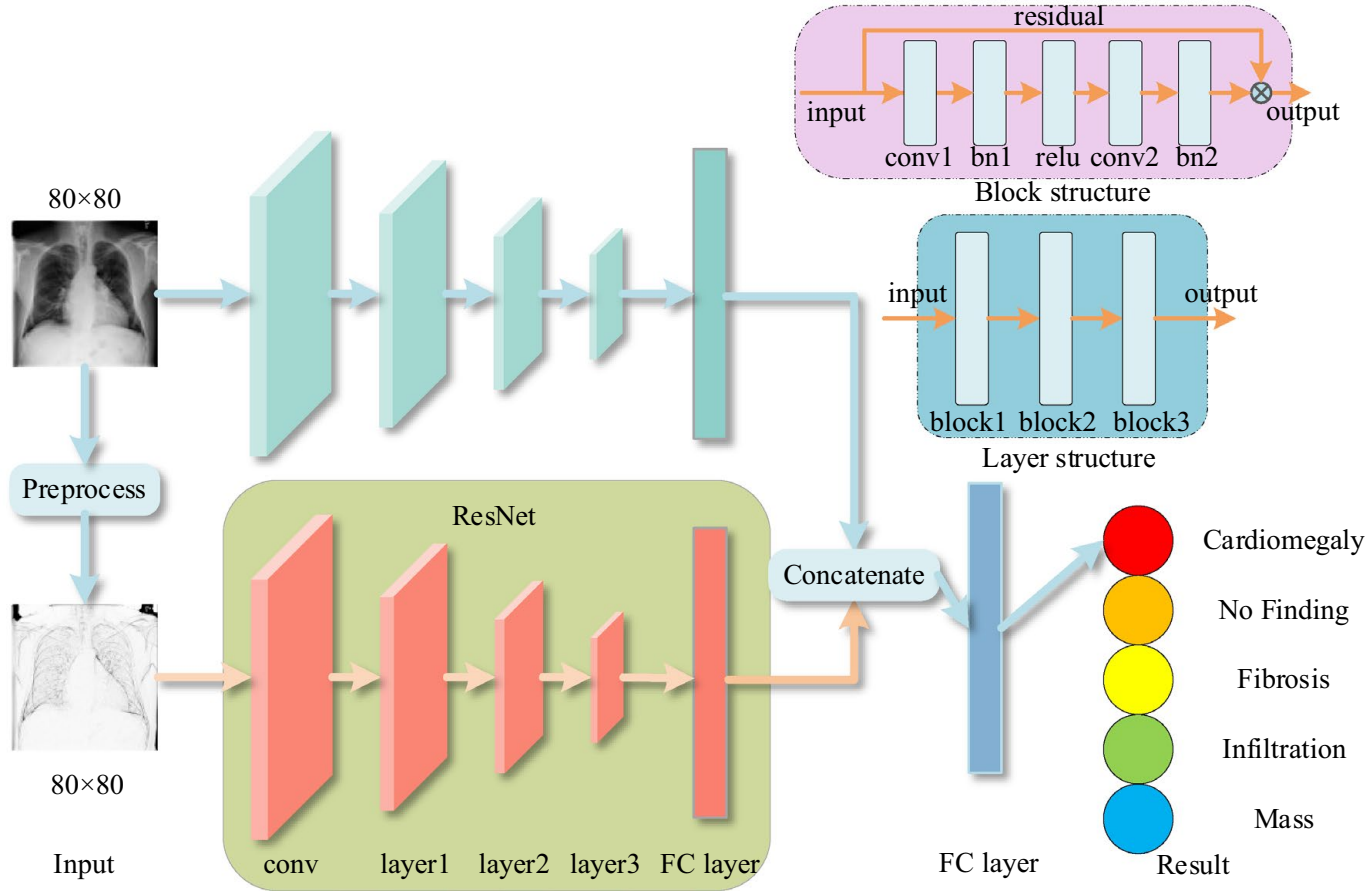
Network structure diagram of the meta-learning process.

The meta-learning phase process is shown in Fig. 3.

In this phase, the weights of the branch network are inherited from the pre-training stage. The fully connected layers are not inherited, but reconstructed and trained. Unlike the pre-training stage, the fully-connected layer of the branch does not make predictions, but instead obtains feature vectors. The feature vectors obtained by the two branches are spliced and predicted by the last fully connected layers. The pre-training process does not use the fully connected layers to integrate the classification results, but trains the two branches as separate networks, and only connects them through the loss function. With this training method, both branches will have certain separate classification capabilities. In the meta-learning, the feature extraction of the two branches is adjusted through the fully connected layers to preserve the recognition ability of the branches as much as possible, so as to be more suitable for cross-domain work.

The meta-test phase adjusts the model parameters with partial data $\{ (\hat{x}_{j} ,\hat{y}_{j} )\}_{j = 1}^{Nt}$ of the target domain $T$ and uses the trained model to predict the classification of the test samples.

## Experimental results and analysis

### Dataset

We use mini-ImageNet^19^ as the source domain and EuroSAT^21^ and ChestX^20^ as the target domain. Due to their large inter-domain differences with mini-ImageNet, they are selected as datasets for cross-domain experiments.

Mini-ImageNet^19^ was proposed by Vinyals et al. for few-shot learning evaluation. Compared to the full ImageNet dataset, it requires fewer resources while maintaining a high level of complexity. The dataset has a total of 100 categories, each with 600 color image samples. The 100 categories are divided into three groups of 64, 16, and 20 for the sampling tasks of meta-training, meta-validation, and meta-testing respectively.

The EuroSAT^21^ dataset is based on Sentinel-2 satellite imagery, covers 13 spectral bands, consists of 10 categories, and contains 27,000 labeled and georeferenced samples. This article uses only the optical RGB bands that are encoded as JPEG images. EuroSAT images are not very similar to those from mini-ImageNet, as the satellite images do not have perspective distortion, but are still color images of natural scenes.

The ChestX^20^ dataset includes 112,120 frontal chest X-ray PNG images at 1024 × 1024 resolution and metadata for all images: image index, lookup label, follow-up number, patient ID, patient age, patient gender, view position, original image size and original image pixel pitch; bounding box for about 1,000 images. Only images with a single disease are used in this article. ChestX images are the least similar to the samples on mini-ImageNet, as they lose perspective distortion, do not represent natural scenes, and only have one color channel.

### Experimental setup

The size of all samples in the experiment is scaled to 92 × 92. The weights of the pre-training loss function are set as $\chi = 1$, $\delta = 1$. The pre-processing gradient scaling are set as $d = 10$. We evaluated our model under 1-shot and 5-shot on the source domain test set, and under 5-way 1-shot, 5-way 5-shot, 5-way 20-shot on the cross-domain test sets. The code is completed under the Pytorch framework. The backbone is ResNet-18. In pre-training phase, mini-ImageNet is used for pre-training, SGD is used as the optimizer, learning rate is set to 0.1, gamma is set to 0.2, step size is set to 30. In meta-learning phase, mini-ImageNet is used for meta-learning, Adam^34^ is used as the optimizer, learning rate are set to 0.001 for classifier and 0.0001 for feature extractor, gamma is set to 0.5, step size is set to 10. The meta-test phase settings are the same as the meta-learning phase.

In addition, we also compare CDPSN without the branch fully-connected layer, which uses the same network as the meta-learning process during network pre-training. It does not contain branched fully connected layers, and the rest is consistent with the CDPSN meta-learning structure.

### Experimental results

Table 1 shows the results that the source domain is mini-ImageNet and the target domains are ChestX and EuroSAT. EGNN^35^, IGN^10^ and MTL^25^ are cross-domain algorithms that have performed well in recent years. MatchingNet^19^, MAML^3^, ProtoNet^6^, RelationNet^4^, MetaOpt^5^ are the classic algorithms for few-shot learning. The optimal values are marked in bold. Where FWT stands for feature-wise transform method^36^. CDPSN (without branch FC layers) method achieves the best performance with 25.26% under 5-way 5-shot setting and with 29.36% under the 5-way 20-shot setting , better than MAML (27.53%), ProtoNet (28.21%) and MTL (28.26%) algorithms. But CDPSN (with branch FC layers) performs poorly. This is due to the similarity of the samples in the ChestX dataset. Changes in bone shape will affect the accuracy. At the same time, because CDPSN (with branch FC layers) increases the influence of the contour by classification through the separate fully connected layers in the pre-training process, the impact of bone changes on the accuracy rate will further increase. Therefore, compared with other algorithms, the accuracy rate is lower. But CDPSN (without branch FC layers) preforms well, which confirms the effectiveness of the preprocessing method. There are still some problems with branch fully connected layers and training strategies when the sample similarity is high. This problem also occurs in other datasets with small differences between categories, such as Cars^37^ and CUB^15^.

**Table 1:** Compare the classification accuracy (%) of different methods on cross-domain test sets.

Methods	ChestX			EuroSAT		
	5-way 1-shot	5-way 5-shot	5-way 20-shot	5-way 1-shot	5-way 5-shot	5-way 20-shot
MatchingNet^19^	–	22.40 ± 0.70	23.61 ± 0.86	–	64.45 ± 0.63	77.10 ± 0.57
MatchingNet + FWT^36^	–	21.26 ± 0.31	23.23 ± 0.37	–	56.04 ± 0.65	63.38 ± 0.69
MAML^3^	–	23.48 ± 0.96	27.53 ± 0.43	–	71.70 ± 0.72	81.95 ± 0.55
ProtoNet^6^	–	24.05 ± 1.01	28.21 ± 1.15	–	73.29 ± 0.71	82.27 ± 0.57
ProtoNet + FWT^36^	–	23.77 ± 0.42	26.87 ± 0.43	–	67.34 ± 0.76	75.74 ± 0.70
RelationNet^4^	–	22.96 ± 0.88	26.63 ± 0.92	–	61.31 ± 0.72	74.43 ± 0.66
RelationNet + FWT^36^	–	22.74 ± 0.40	26.75 ± 0.41	–	61.16 ± 0.70	69.40 ± 0.64
MetaOpt^5^	–	22.53 ± 0.91	25.53 ± 1.02	–	64.44 ± 0.73	79.19 ± 0.62
EGNN^35^	–	–	–	–	69.35 ± 0.79	–
ResNet-18^38^	22.46 ± 0.42	24.68 ± 0.45	–	57.54 ± 0.94	74.40 ± 0.74	–
MTL(ResNet-25)^25^	**22.55 ± 0.40**	24.73 ± 0.45	28.26 ± 0.46	**60.57 ± 0.94**	78.12 ± 0.74	83.23 ± 0.64
IGN^10^	–	–	–	–	79.78 ± 0.83	–
CDPSN (with branch FC layers)	22.12 ± 0.39	24.86 ± 0.42	27.11 ± 0.38	57.16 ± 0.90	**81.52 ± 0.66**	**86.98 ± 0.51**
CDPSN (without branch FC layers)	22.54 ± 0.38	**25.26 ± 0.41**	**29.36 ± 0.46**	60.29 ± 0.90	77.93 ± 0.69	81.50 + 0.60

On the EuroSAT dataset, CDPSN (with branch FC layer) achieves the best results on both 5-shot with 81.52% and 20-shot with 86.98%, 1.74% ahead of second place IGN in 5-way 5-shot and 3.75% ahead of second place MTL in 5-way 20-shot. At the same time, it is better than excluding branch fully connected layers on 5-way 5-shot and 5-way 20-shot. This is due to the large difference in contours between sample categories in the EuroSAT dataset, and CDPSN (with branch FC layers) that emphasizes contours can classify more accurately.

CDPSN (with branch FC layers) underperforms under all 5-way 1-shot conditions This is also caused by the training strategy. Under the 1-shot condition, it is difficult for the weights inherited from pre-training to change from independent prediction to cooperative prediction. But when the training samples are sufficient, the task can be completed with higher accuracy by CDPSN.

Table 2 shows the evaluation results on the source domain mini-ImageNet, and the optimal values are marked in bold. The results show that CDPSN (with branch FC layers) still performs poorly under the 1-shot condition, but under the 5-shot condition, while using the generalization strategy, CDPSN (with branch FC layers) still guarantees the accuracy of the source domain at 80.68%. This result is higher than 79.94% of IGN and 80.51% of CTM.

**Table 2:** Compare the classification accuracy (%) of different methods on source domain test set.

Methods	mini-ImageNet	
	5-way 1-shot	5-way 5-shot
Delta-encoder^39^	58.7	73.6
MatchingNet^19^	43.44 ± 0.77	55.31 ± 0.73
ProtoNet^6^	49.42 ± 0.78	68.20 ± 0.66
RelationNet^4^	50.44 ± 0.82	65.32 ± 0.70
TADAM^40^	58.5 ± 0.3	76.7 ± 0.3
MAML^3^	48.70 ± 1.75	63.11 ± 0.92
MetaOpt^5^	62.64 ± 0.35	78.63 ± 0.68
CTM^41^	64.12 ± 0.82	80.51 ± 0.13
LGM-Net^42^	**69.13 + 0.35**	71.18 + 0.68
ResNet-18^38^	59.88 ± 0.88	75.71 ± 0.65
MTL (ResNet-18)^25^	61.7 ± 1.8	75.6 ± 0.9
IGN^10^	-	79.94 ± 1.05
CDPSN (with branch FC layers)	58.65 ± 0.92	**80.68 ± 0.66**
CDPSN (without branch FC layers)	60.84 ± 0.97	77.69 ± 0.68

**Figure 4:**
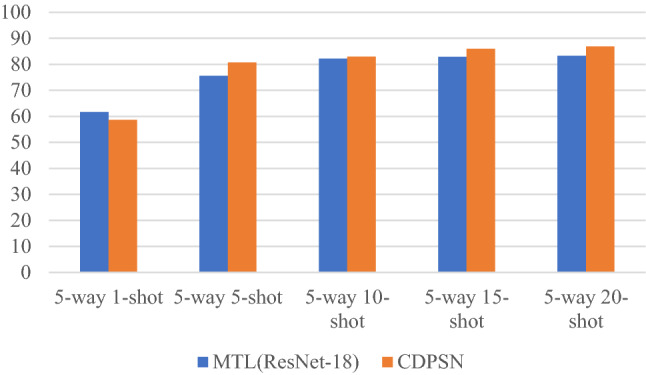
Histogram of accuracy change on mini-ImageNet per 5-shot compared with MTL.

Table 3 and Fig. 4 show the accuracy changes on mini-ImageNet per 5-shot increase compared with MTL. As shown in Table 3 and Fig. 4, except the result on 1-shot, the rest of the results are better than MTL. When the number of samples increases from 1 to 5, the accuracy of CDPSN is significantly improved by 22.03%, and as the number of samples increases, the growth rate of the accuracy is still higher than that of the MTL algorithm.

**Table 3:** Classification accuracy (%) changes on mini-ImageNet per 5-shot compared with MTL.

	5-way 1-shot	5-way 5-shot	5-way 10-shot	5-way 15-shot	5-way 20-shot
MTL (ResNet-18)	61.7	75.6	82.18	82.88	83.25
CDPSN (Ours)	58.65	80.68	82.94	85.98	86.88

### Preprocessing ablation experiments

**Figure 5:**
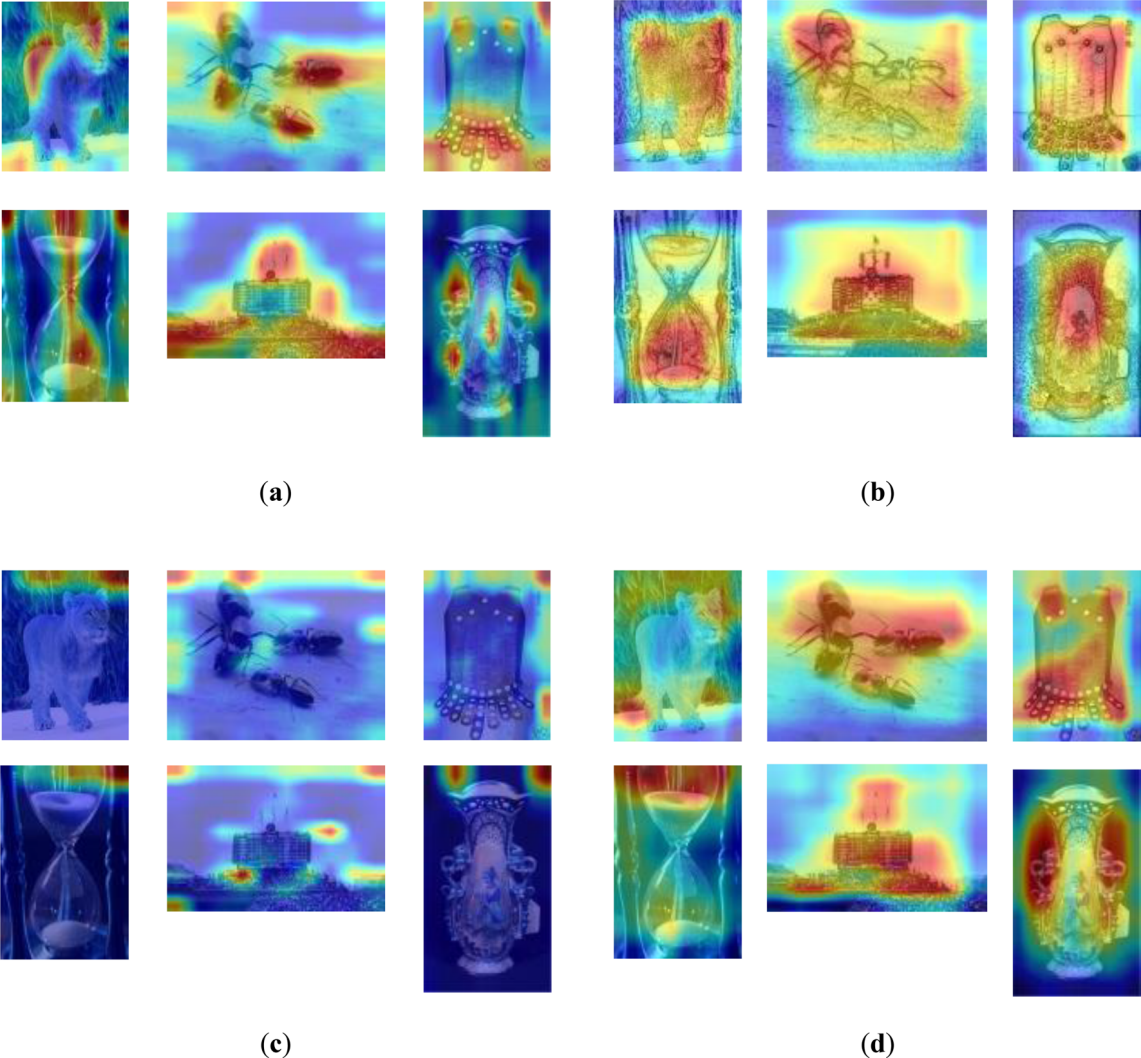
Grad-CAM++ heatmaps under 5-way 5-shot in preprocessing ablation experiments. (**a**) heatmap of original branch of CDPSN; (**b**) heatmap of sketch branch of CDPSN; (**c**) heatmap of original branch without sketch preprocessing; (**d**) heatmap of sketch branch without sketch preprocessing.

We conduct an ablation experiment to demonstrate how the sketch preprocessing component enhances the source domain accuracy. CDPSN (without preprocess) still has two branches, but the preprocessing part is deleted, and only the original samples are used to learn in the two branches. The rest part is the same as CDPSN. The results are shown in Table 4 and Fig. 5. The images in Fig. 5 are from mini-ImageNet dataset. Table 4 shows that sketch preprocessing part improve the accuracy of the network by 4.18% under 5-way 1-shot setting, and 5.52% under 5-way 5-shot setting, compared with the network without sketch preprocessing. As can be seen from Fig. 5, Fig. 5a is the heatmaps of original branch of CDPSN. The original branch learns the detail of samples, while the sketch branch, heatmaps shown by Fig. 5b, learns the parts with obvious contour lines. Although CDPSN (without preprocess) has also learned classification features, one of the branches does not learn the correct features. Only one branch is supporting the whole network. The accuracy 75.16% of CDPSN (without preprocess) is even lower than the accuracy 75.71% of ResNet under the 5-way 5-shot condition, which means the branch in Fig. 5c not only did it not play a positive role, but it lowered the accuracy due to learning incorrect features. Compared with the network without sketch preprocessing, the two branches of CDPSN have a more obvious division of labor. The sketch branch of CDPSN can capture global information better, and can better extract contour information, while original branch can extract local key information better.

**Table 4:** Classification accuracy (%) in preprocessing ablation experiments on mini-ImageNet.

	5-way 1-shot	5-way 5-shot
CDPSN	58.65 ± 0.92	80.68 ± 0.66
CDPSN (without preprocess)	54.47 ± 0.84	75.16 ± 0.69

## Feasibility qualitative analysis based on heat map

We use Grad-CAM++^43^ as a tool for generating heat maps. Grad-CAM^44^ can obtain the gradient of the feature map according to the output vector and obtain the gradient map corresponding to each feature map. Then average the gradient maps to obtain the features. The class activation graph is finally created by the activation function after the weights of the graph are weighted and summed with the feature graphs. Compared with Grad-CAM, Grad-CAM++ adds an extra weight to weight the elements on the gradient map so that each element on the gradient map contributes differently. All images used below are from the mini-ImageNet dataset and EuroSAT dataset.

**Figure 6:**
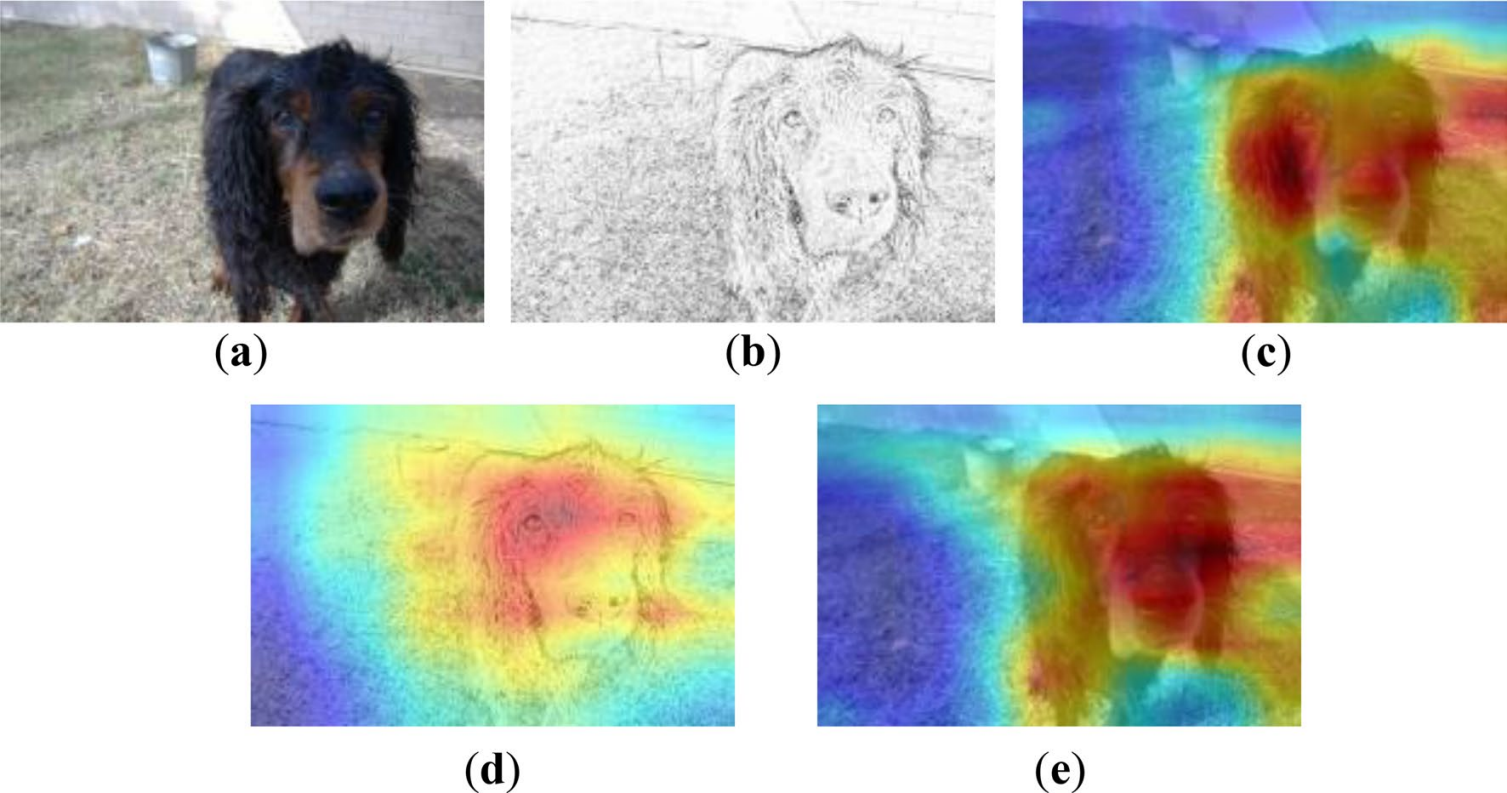
Grad-CAM++ heatmaps after pre-training phase of CDPSN and MTL. (**a**) original input image; (**b**) preprocessed image; (**c**) heatmap of original branch of CDPSN; (**d**) heatmap of sketch branch of CDPSN; (**e**) heatmap of MTL network.

The heatmaps after pre-training is shown in Fig. 6, and the target layer of the heatmaps is the last convolutional layer. Compared with the MTL network, CDPSN uses the original feature extraction branch to identify the key features of image details, and sketch branch to extract large-scale contour features from the image, which can better scan contour features and complete classification information.

**Figure 7:**
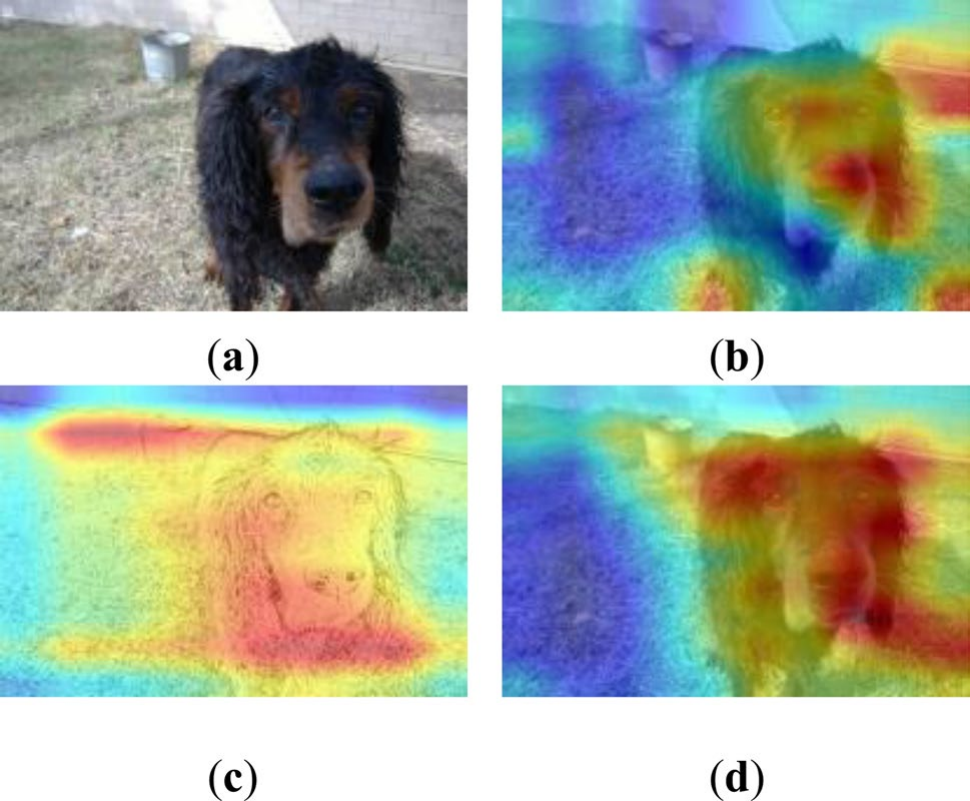
Grad-CAM++ heatmaps under 5-way 1-shot after meta-learning phase of CDPSN and MTL. (**a**) original input image; (**b**) Heatmap of original branch of CDPSN; (**c**) heatmap of sketch branch of CDPSN; (**d**) heatmap of MTL network.

The heatmaps after meta-learning is shown in Fig. 7. The target layer of the heatmaps is the last convolutional layer. The meta-learning phase of CDPSN network increases the attention to contour information and weakens the influence of details on classification. The original feature extraction branch pays more attention to the local information important for classification, and the contour information is extracted by the sketch branch. Compared with the original feature extraction branch, since the preprocessed image contains relatively little feature information, there may even be blank areas, the contour information can be seen more clearly in the sketch branch. Compared with the heatmaps of the MTL network, CDPSN pays more attention to the contour, which provides a basis for cross-domain generalization. While the original feature extraction branch pays less attention to the information than the MTL network, and focuses more on local information, The capture of contour information by sketch branch mitigates the adverse effects.

**Figure 8:**
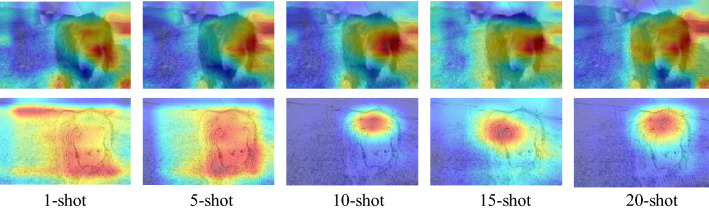
Changes in the heatmaps of CDPSN under 1–20 shots.

The heatmaps changes of CDPSN under 1–20 shots are shown in Fig. 8. As can be seen in Fig. 8, in the case of insufficient number of shots, in order to better generalize the network, the sketch branch of CDPSN tends to global search to ensure that information is not missed, which also leads to lower accuracy under 1-shot condition. When the number of shots reaches 5, the sketch branch can basically delineate the effective information. As the number of shots increases, the information that needs to be learned for classification is more accurately captured, leads to the decrease of the importance of focusing on global information, the focus of the sketch branch tends to converge. The sketch branch pays local attention to valid information more accurately, while the remaining detailed semantic information is supplemented by the original branch, which can classify the samples more accurately. This shows the feasibility of CDPSN.

**Figure 9:**
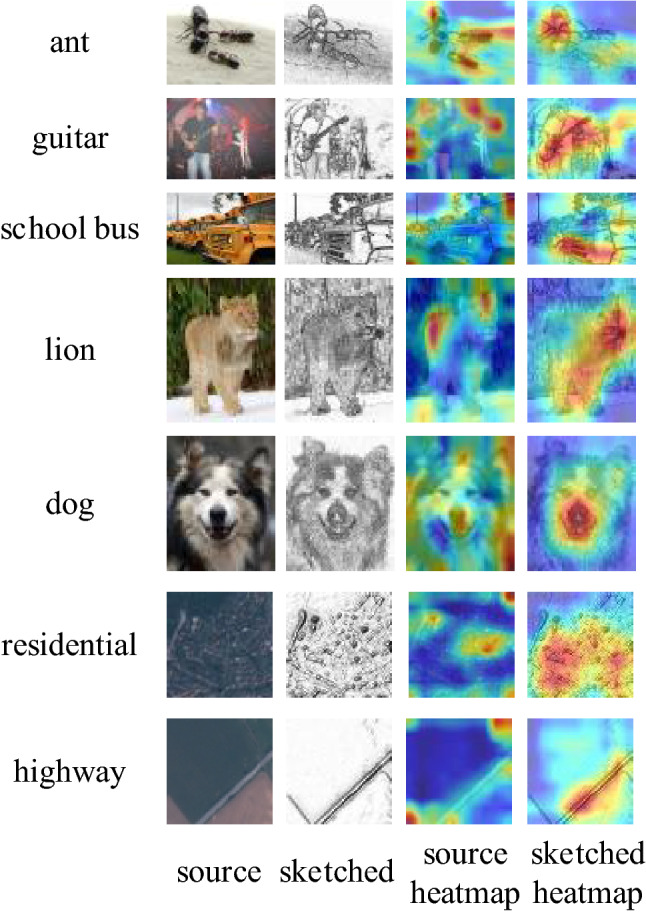
The heatmaps of CDPSN under 10-shot on mini-ImageNet and EuroSAT.

For the target domain that is significantly different from the source domain, CDPSN can capture the entire effective contour information when the number of shots is not enough. Unlike domain alignment methods, for the sketch branch, there is no inter-domain difference in color and channel effects, only influenced by of perspective distortion and contour discrimination. Therefore, CDPSN method performs better on EuroSAT, even better than the source domain mini-ImageNet. The heatmaps of different categories of CDPSN under 10-shot is shown in Fig. 9.

## Conclusion

By analyzing the existing algorithms of cross-domain few-shot learning, we propose a cross-domain few-shot learning method based on pseudo-Siamese neural network. By simultaneously learning the original image and the preprocessed image, the key contour feature can be better captured in the cross-domain process. While the feasibility of the model is illustrated by the heat map, the experimental results on the EuroSAT and ChestX public datasets as the cross-domain target domain and the mini-ImageNet public dataset as the source domain also show that this method shows good generalization ability for tasks with large domain differences. This advantage is particularly noticeable when there are significant class contour distinctions. While verifying the generalization performance of the model, we also verify in the source domain that the model does not reduce the classification accuracy of the source domain due to generalization. However, CDPSN does not perform well in cases where the difference between classes is not large. The preprocessing method used by CDPSN will generate certain noise, and the use of filtering will destroy details such as texture, and will greatly reduce the efficiency. At the same time, in the process of sample normalization, the mean and variance of the sketch map are the same as those of the natural image, which may be unreasonable. The training process also takes more time due to the use of a pseudo-Siamese neural network structure. In the future, we will start from the two directions of improving efficiency and focusing on local differences, and explore more reasonable pre-training strategies to integrate the advantages of CDPSN with branch fully connected layers and CDPSN without branch fully connected layers.

## Editorial board members and editors

The author has no competitive interest with members of the Editorial Committee and Editors.

## Data Availability

The datasets used in this research work are publicly available and can be downloaded from the website below. Mini-ImageNet: https://github.com/vieozhu/MAML-TensorFlow-1. EuroSAT: https://github.com/phelber/EuroSAT. Chestx: https://nihcc.app.box.com/v/ChestXray-NIHCC.
